# The Relationship between Impulsivity and Problem Gambling in Adolescence

**DOI:** 10.3389/fpsyg.2016.01931

**Published:** 2016-12-08

**Authors:** Roberto Secades-Villa, Victor Martínez-Loredo, Aris Grande-Gosende, José R. Fernández-Hermida

**Affiliations:** Department of Psychology, University of OviedoOviedo, Spain

**Keywords:** adolescence, gambling, impulsivity, delay discounting, risk factors

## Abstract

Gambling has become one of the most frequently reported addictive behaviors among young people. Understanding risk factors associated with the onset or maintenance of gambling problems in adolescence has implications for its prevention and treatment. The main aim of the present study was to examine the potential relationships between impulsivity and problem gambling in adolescence. Participants were 874 high school students (average age: 15 years old) who were surveyed to provide data on gambling and impulsivity. Self-reported gambling behavior was assessed using the South Oaks Gambling Screen – Revised for Adolescents (SOGS-RA) and impulsivity was measured using the Impulsive Sensation Seeking Questionnaire (ZKPQ), the Barratt Impulsiveness Scale (BIS-11-A), and a delay discounting task. The data were analyzed using both a prospective-longitudinal and a cross-sectional design. In the longitudinal analyses, results showed that the impulsivity subscale of the ZKPQ increased the risk of problem gambling (*p* = 0.003). In the cross-sectional analyses, all the impulsivity measures were higher in at-risk/problem gamblers than in non-problem gamblers (*p* = 0.04; 0.03; and 0.01, respectively). These findings further support the relationship between impulsivity and gambling in adolescence. Moreover, our findings suggest a bidirectional relationship between impulsivity and problem gambling in adolescence. These results have consequences for the development of prevention and treatment programs for adolescents with gambling problems.

## Introduction

There is growing evidence that opportunities to gamble and problematic gambling among adolescents are increasing in developed countries, mainly due to the growing availability of and access to online gambling (Huang and Boyer, 2007; Blinn-Pike et al., 2010; Secades-Villa et al., 2014). Problem gambling in young people has been associated with significant psychosocial and mental health problems, such as disruptive family relationships (Hardoon et al., 2004), school failure (Potenza et al., 2011), conduct and substance misuse problems (Hardoon et al., 2004; Estevez et al., 2015), depression (Hardoon et al., 2004; Lynch et al., 2004; Estevez et al., 2015), or ADHD (Derevensky et al., 2007).

In addition to the structural characteristics of the gambling activity itself (Parke and Griffiths, 2007), there are several potential individual and social factors contributing to the development and maintenance of problem gambling (Donati et al., 2013; Dixon et al., 2016). However, to date, very few studies have focused on risk factors that contribute to engagement in problem gambling in adolescent samples (Cosenza and Nigro, 2015).

As regards individual determinants, impulsivity seems to be one of the most critical factors associated with problem gambling and other disorders in adolescence such as substance use problems (Pérez-Fuentes et al., 2015) or eating disorder behavior (Wonderlich et al., 2004). Impulsivity is a multidimensional construct often defined as a human behavior characterized by the inclination of an individual to act on urge rather than thought, with diminished regard to consequences, and encompasses a range of maladaptive behaviors which are in turn affected by distinct neural systems (Meda et al., 2009). Impulsivity has been found to increase the likelihood of gambling onset in youths with low – but not high – socio-economic status (SES) (Auger et al., 2010; Dussault et al., 2011), and to predict gambling frequency (Benson et al., 2012) and problem gambling in low SES adolescent males. One study also found that positive urgency was associated with stronger scores of both gambling frequency and problem gambling (Canale et al., 2016).

Despite the significant contributions from previous studies, important questions remain regarding the influence of impulsivity on gambling severity and gambling onset among young people (Cosenza and Nigro, 2015). For example, almost all studies used cross-sectional designs (Barnes et al., 2005; Leeman et al., 2014; Canale et al., 2016) that cannot address directionality, and most of the few longitudinal studies only included male subjects (Vitaro et al., 1997, 1999; Dussault et al., 2011; Liu et al., 2013). Several previous studies did not find a relationship between impulsivity and gambling among young people (Barnes et al., 2005; Lee et al., 2011). Finally, most of the research has focused on only one of the domains of impulsivity (Mackillop et al., 2014; Cosenza and Nigro, 2015), evaluated in most cases with self-reports, and we do not know if the findings are generalizable to other impulsivity measures, including behavioral ones (Auger et al., 2010). To our knowledge, only three studies (all of them cross-sectional) in non-clinical samples have analyzed the relation between gambling and delay discounting among adolescents, and they have produced contradictory results (Holt et al., 2003; MacKillop et al., 2006; Cosenza and Nigro, 2015).

Thus, we sought to build on previous research to examine the relationship between different impulsivity domains (including both survey assessments and behavioral tasks) and gambling severity in a representative community sample of adolescents, using both a cross-sectional and a prospective longitudinal design. Given the findings summarized above, we hypothesized that the relationship between impulsivity and problem gambling in adolescence is bidirectional and that all impulsivity domains are related to gambling severity.

## Materials and Methods

### Participants

The participants were 1,327 adolescents aged between 14 and 17 years, recruited from a total of 16 Spanish secondary schools in the region of Asturias. The schools were selected following a random stratified and incidental procedure. The inclusion criteria were: (1) having no sensory impairment, (2) not presenting difficulties understanding the Spanish language, and (3) not being diagnosed with an intellectual disability. Of the initial participants, 1,249 met the inclusion criteria for the cross-sectional analyses. The longitudinal analyses were performed 2 years later with a sub-sample of 874 respondents (56.1% males and 43.9% females). None of the participants refused to be assessed and participants were given guarantees of total confidentiality and anonymity. Participants’ characteristics are shown in **Table 1**.

**Table 1:** Sample characteristics.

	Total sample (*n* = 874)	Non-problem gamblers (*n* = 832)	At-risk and problem gamblers (*n* = 42)	*p*	Effect size
Age (years)^a^	14.93 ± 0.53	14.93 ± 0.52	14 ± 0.66	0.387^1^	–
Gender (% males)	56.1	55	76.2	0.011^2^	0.091^∗^
Amount of weekly allowance (% participants between 20 and 40€)	8	7.9	9.5	0.04^3^	0.123^∗∗^
Living with parents (% yes)	77.1	76.2	69	0.383^2^	–
Employment situation mother (%full time)	39.4	40	26.2	0.190^2^	–
Employment situation father (%full time)	53.8	53.7	54.8	0.153^3^	–
Relatives with problem gambling (% no)	98.4	98.2	97.6	1^2^	–

*^a^Mean ± Standard deviation; ^1^Student *t-*test; ^2^Yates correction for continuity; ^3^Chi-square; ^∗^Phi; ^∗∗^Cramer’s V.*

### Procedure

After the acceptance of participation from schools, students were surveyed in their own classroom using digital devices (Samsung Galaxy Tab2 10.1 tablet). This method was used with the aim of reducing inconsistent answers. The software did not allow participants to skip any questions and was designed to avoid the asking of inappropriate questions in accordance with previous answers. Participants completed the battery of tests, which took a maximum of 75 min, sitting at individual desks, with supervisors checking that they were doing the task appropriately and making sure there was no interaction between them. The survey was designed in such a way that it allowed the individualization of the questions asked as a function of previous answers given by each participant. Before the start of the assessment, trained experimenters provided instructions on how to perform the tasks. Participants were also assured of complete anonymity and confidentiality.

### Measures

#### Demographic Data

Data was collected regarding age, gender, number of siblings, amount of weekly allowance, family structure (i.e., living with no parents or with one or two parents), and the employment status of parents. Information on whether the participants had relatives with problematic gambling habits was also gathered.

#### Gambling Behavior

Participants completed a survey about their gambling activities, both in land-based and online-based modes. Gambling was defined as “any game that involves betting with money.” The following gambling activities were measured: bingo, poker, other casino games (OCGs), sports betting, lottery, scratch-tickets, and electronic gambling machines (EGMs). Participants indicated how often they had engaged in each of these activities throughout their lifetimes, over the past year, and over the past month. Participants also indicated: age of gambling onset, time spent gambling, amount of money wagered on a regular day of gambling, and company (if they gambled alone or with other people).

Adolescent gambling behavior was also measured with the South Oaks Gambling Screen – Revised for Adolescents (SOGSRA) (Winters et al., 1993); Spanish version (Becona, 1997). It consists of 10 dichotomous items (no = 0, yes = 1) assessing gambling behavior and gambling-related problems during the past 12 months. The total score ranges from 0 to 12. Scores provide three categories: non-problem gambler (score of 0 or 1), at-risk gambler (score of 2 or 3) and problem gambler (score of 4 or more). The Spanish version yielded a Cronbach’s alpha of 0.80.

Three different measures of impulsivity were measured due that reveal independent domains of impulsivity that are related to gambling severity:

The Zuckerman-Kuhlman Personality Questionnaire (ZKPQ) (Zuckerman et al., 1993), which consists of true/false questions, eight of which pertain to impulsivity (primarily lack of premeditation) and eleven of which pertain to sensation seeking. For the purpose of this study, we used the impulsivity (Imp) subscale; Spanish version (Fernández-Artamendi et al., 2016). The Spanish version yielded a Cronbach’s alpha of 0.83 (Imp: α = 0.75; SS: α = 0.74).

The Barratt Impulsiveness Scale (BIS-11-A; Patton et al., 1995); Spanish version (Martínez-Loredo et al., 2015). This measure of impulsivity captures the following three domains: (a) Attentional Impulsivity: difficulty dedicating adequate attention to a task; (b) Motor Impulsivity: propensity to act rashly without forethought; and (c) Non-planning Impulsivity: failure to adequately plan ahead. It contains 30 descriptive statements (maximum score of 120) to which participants respond with the extent to which each statement describes them on a 4-point Likert scale (1 = rarely/never; 2 = occasionally; 3 = often; 4 = almost always/always). The BIS-11-A consists of two subscales: general impulsivity (BIS-g) and non-planning impulsivity (BIS-np). Its validation with Spanish adolescents showed a good reliability with a Cronbach’s alpha of 0.87 (Cronbach’s alpha = 0.91 for BIS-g; and 0.85 for BIS-np).

#### Delay Discounting

Delay discounting describes how a reinforcer loses value as the delay to its receipt increases (Bickel and Marsch, 2001). Delay discounting is typically assessed using an adjusting-delay procedure in which an individual is presented with multiple choices (usually, hypothetical monetary rewards) between a smaller, more immediate reinforcer vs. a larger, more delayed one. The delay discounting task was presented to participants via a tablet running Android 4.0.3 operating system. Overall, the task took approximately 10 min to complete for each participant. Participants were instructed on how to interact with the delay discounting program and informed that they would not receive any of the monetary amounts presented, but that they were to respond as if the choices were real. Previous studies have demonstrated that participants respond similarly during delay discounting tasks for both real and hypothetical monetary values.

Participants were presented with a choice between €1,000 after a fixed delay, versus various amounts of money available immediately using an adjusting-amounts procedure (Holt et al., 2012). The delays values were 1 day, 1 week, 1 month, 6 months, 1 year, 5 years, and 25 years. The delays were presented in an ascending order for all the participants. The value of the immediate monetary option ranged from €5 to €1,000 in €5 increments and was adjusted via a titrating procedure that honed in on the indifference point based on the participants’ responses. The titration procedure took the lower and upper limit of possible values (initial €0 and €1,000) and divided this total range randomly by 2, 3, or 4 to obtain an interval value. The value of the immediate option was one interval value above or below the upper and lower limits. If the immediate value was outside €0 and €1,000, another value was randomly chosen. New lower and upper limits were chosen based on the participant’s response, adjusting the total range, and the titration process was repeated. Note that based on the possible values presented, the total range could occasionally increase if they chose an option outside of the total range. Once the total range was at or less than €40, the average of the upper and lower limits was taken as the indifference point, and the next delay was presented.

The dependent variable was the *k*-value, which describes the rate of discounting, with higher *k*-values showing greater discounting and more impulsive choices selected. In order to assess *k*-values for each participant, individual indifference points were fitted to the hyperbolic equation described by Mazur (1987):

$$\begin{array}{c} V\;=\;A/(1+kD) \end{array}$$

The Eq. (1) shows how the value (*V*) of a reward of certain amount (*A*) is discounted as a function of delay (*D*) to its delivery (Mazur, 1987). As the distribution of *k*-values was skewed, analyses were performed on log-transformed *k*-values.

#### Control Variables

With the aim of detecting random answers, an infrequency scale was used (Oviedo Infrequency Scale, INF-OV) (Fonseca-Pedrero et al., 2009). This instrument is composed of 12 items randomly interspersed and mixed throughout the assessment. Participants were required to respond to five-level Likert-type items (from totally disagree to totally agree) about obvious facts such as ‘I know people who wear glasses’ or ‘I have sometimes watched films on TV.’ The total score ranged from 0 to 60 points, with participants scoring more than three points on the scales being removed.

### Data Analyses

Various descriptive and frequency analyses were carried out in relation to the participants’ characteristics. Due to the low sample size in each SOGS-RA category and the substantial problems already associated with both at-risk and problem gambling among adolescents (Potenza et al., 2011), participants were classified in two groups on the basis of their score on the SOGS-RA: Non-problem gamblers (SOGS-RA ≤ 1; *n* = 282) versus at-risk and problem gamblers (SOGS-RA ≥ 2; *n* = 42). In order to analyze the association between impulsivity and problem gambling, Pearson’s correlation between SOGS-RA and impulsivity scores were performed. On the other hand, to test previous impulsivity differences on adolescents with and without gambling problems, longitudinal analysis were performed using impulsivity scores obtained in the first wave and gambling score in the second wave. Also, a cross-sectional analysis was performed using impulsivity and gambling scores, both from the second wave. Several Student’s *t*-tests were applied to test the relationship between the three different measures of impulsivity and problem gambling in both longitudinal and cross-sectional analyses. Effect sizes of principal comparisons were calculated using Cohen’s d (*d*), due to the discrepancy between group sizes (Field, 2007). Values for small, medium and large effects for eta squared are 0.01, 0.06, and 0.14, respectively. Confidence level was 95%, and the statistical package used was the SPSS (V20; SPSS, Inc., Chicago, IL, USA).

## Results

### Gambling Prevalences

Overall, 55.3% (*n* = 483) of participants reported gambling behavior during their lifetime, 37.1% (*n* = 324) of participants reported gambling in the past year, and 24% (*n* = 210) in the last month. On the basis of their scores on the SOGS-RA, 95.2% (*n* = 282) of the total sample were non-problem gamblers, 1.1% (*n* = 10) were problem gamblers and 3.7% (*n* = 32) were at-risk gamblers.

### Gambling Characteristics

The most common gambling activities were the lottery (47.4%), sport betting (38.2%), scratch-cards (34.5%), poker (21.8%), bingo (28.6%) and EGMs (3.1%).

Regarding the mode of access (i.e., land-based, online-based and mixed-mode): 87.7% (*n* = 285) reported only land-based (non-internet) gambling, 0.9% reported only online-based gambling (*n* = 3), and 11.4% (*n* = 37) reported both non-internet and online-based gambling.

### Gambling and Impulsivity

Relationships between all the impulsivity measures and gambling severity are presented in **Table 2**. All measures were significantly correlated with SOGS-RA scores excepting log*k* at the first wave. Mean differences in impulsivity according to problem gambling are shown in **Table 3**. In the longitudinal analyses, participants with high scores on the ZKPQ impulsivity subscale were more likely to be at-risk or problem gamblers in the second wave (*p* = 0.003). In the cross-sectional analyses, participants with high scores on all the impulsivity measures (ZKPQ imp subscale, BIS-11-A and log*k*) were more likely to be at-risk or problem gamblers.

**Table 2:** Correlation between impulsivity measures and gambling severity.

	South Oaks Gambling Screen
BIS-11-A_1_	0.138^∗^
BIS-11-A_2_	0.138^∗^
Impulsive Subscale_1_	0.195^∗∗^
Impulsive Subscale_2_	0.216^∗∗^
Log*k*_1_	-0.039
Log*k*_2_	0.182^∗∗^

*BIS, Barratt Impulsiveness Scale; Log*k*, log-transformed *k-*value; subindices represent assessment waves. ^∗^*p* < 0.05; ^∗∗^*p* < 0.001.*

**Table 3:** Impulsivity by gambling severity.

	Longitudinal analyses					Cross-sectional analyses				
	Non-problem gamblers	At-risk and problem gamblers				Non-problem gamblers	At-risk and problem gamblers			
	*M* ±*SD*	*M* ±*SD*	*t*_df_	*p*	*d*	*M* ±*SD*	*M* ±*SD*	*t*_df_	*p*	*d*
Imp	3 ± 2.30	4.17 ± 2.37	-3.037_322_	0.003	0.34	3.23 ± 2.40	4.62 ± 2.22	-3.527_322_	0.001	0.39
BIS-11-A	38.84 ± 15.42	43.52 ± 16.49	-1.818_322_	0.070	-	47.03 ± 10.51	53.95 ± 14.72	-2.935_47.42_	0.005	0.85
log*k*	-2.39 ± 1.47	-2.53 ± 1.53	0.587_322_	0.558	-	-2.74 ± 1.17	-2.32 ± 1.54	-2.071_322_	0.039	0.23

*M ± SD, Mean ± Standard deviation; Imp, Impulsive Subscale (Zuckerman-Kuhlman Personality Questionnaire; ZKPQ); BIS-11-A, Barratt Impulsiveness Scale; log*k*, log-transformed *k-*value.*

## Discussion

The main purpose of this study was to test the relationship between impulsivity and gambling status during adolescence. Results showed that the prevalence of at-risk and problem gambling was 4.8%, that impulsivity precedes later gambling problems, and, significantly, that gambling problems increase impulsivity.

The percentage of at-risk and problem gambling among the total sample of adolescents was substantially lower than those found in previous studies (Olason et al., 2011; Jonkman et al., 2013; Dixon et al., 2016). Similarly, gambling prevalence among those who gambled in the last year was still below that found in previous studies (McCormack et al., 2013; Castren et al., 2015; Canale et al., 2016). Several factors might explain this divergent result. First, the legal restrictions enacted in Spain over the last few years may have contributed to reducing gambling prevalence among adolescents. Second, our study was conducted with a sample of adolescents aged under 18 while the vast majority of previous research included samples with a broad range of ages. Moreover, many studies only report gambling severity rates among bettors instead of the percentages of gambling severity among the total sample (Potenza et al., 2011; Gainsbury et al., 2015).

Different sources of impulsivity measure may contribute to the different findings in the two analyses (longitudinal and cross-sectional): High scores on the ZKPQ impulsivity subscale at the first wave increased the risk of problem gambling at the second wave, and all the impulsivity measures (ZKPQ imp subscale, BIS-11-A and log*k*) were related to gambling problems in the cross-sectional analyses.

Consistent with previous studies (Vitaro et al., 1997, 1999; Dussault et al., 2011; Liu et al., 2013), youths who report a tendency toward impulsive behavior, specifically acting without thinking or planning, may be at risk for problem gambling in adolescence. Several factors may contribute to this result. It is possible that highly stimulating activities, such as gambling, are often pursued as a means to relieve stress among individuals with high impulsivity (Jacobs, 1986). Impulsive individuals tend to be more exposed to excessive chronic stress resulting from a hypo-aroused psychological state (Gupta and Derevensky, 1998). Impulsive youths may be at risk of developing gambling problems due to the fact that gambling often involves a high degree of sensory and mental stimulation (Nower et al., 2004). Finally, immaturity of frontal cortical and subcortical monaminergic systems during neurodevelopment in adolescence may predispose individuals to trait impulsivity, resulting in increased vulnerability to addictive behaviors such as problem gambling (Chambers and Potenza, 2003).

Our results also indicated that beyond the effect of impulsivity on gambling, all the impulsivity measures and tasks were significantly associated with gambling severity in the cross-sectional analyses. Previous studies using both measures of impulsivity, personality inventories (Vitaro et al., 1999; Liu et al., 2013), and delay discounting tasks (Alessi and Petry, 2003; Cosenza and Nigro, 2015) have found that gambling problems increase impulsivity. These results suggest that individuals who have the general tendency to make impulsive monetary decisions may also behave impulsively (Alessi and Petry, 2003). The fact that in these analyses the link between problem gambling and impulsivity is not dependent on a particular measure further supports the validity of this link.

Taken together, our results suggest that the link between impulsivity and problem gambling in adolescence is probably bidirectional, both influencing the other mutually in a negatively interactive spiral. These results are in agreement with research and clinical expertise that consider impulsivity to be integral to understanding pathological gambling behavior (Alessi and Petry, 2003).

This association suggests that these two problems are to be approached jointly when treating problem gambling in adolescents. The significant association of youth impulsivity with subsequent gambling problems highlights the importance of identifying and intervening with impulsive adolescents to prevent adolescent problem gambling. Moreover, the influence of gambling on impulsivity underscores the importance of developing innovative intervention strategies directed at decreasing impulsivity in this population.

This study has several limitations. First, the study sample consisted of urban participants, so the findings may not be generalizable to the general population and thus should be extrapolated with caution. Second, the observed significant association between impulsivity and gambling does not necessarily indicate a causal relationship. Third, self-reports of gambling problems may be subject to reporting bias.

Despite these limitations, the present study provides further evidence on the nature of the relationship between impulsivity and problem gambling. Our study indicates an inter-relationship between these constructs. Impulsivity, in terms of acting without thinking or planning, precedes later gambling problems, and gambling problems increase self-reported impulsivity and the preference for small immediate rewards over larger delayed rewards (delay discounting).

## Ethics Statement

The study was approved by the Ethics Committee of the Spanish Education Ministry. Written consent was obtained from their parents.

## Author Contributions

RS-V designed the study, VM-L and AG-G managed the literature searches and wrote the first draft of the manuscript. JF-H conducted the statistical analyses. All authors contributed to and have approved the final manuscript.

## Conflict of Interest Statement

The authors declare that the research was conducted in the absence of any commercial or financial relationships that could be construed as a potential conflict of interest.
